# A Temperature Measurement and System Identification Method for Confined Cavity Explosions Based on an Improved Type C Thermocouple Sensor

**DOI:** 10.3390/s26061948

**Published:** 2026-03-20

**Authors:** Zhaoxiang Niu, Jijun Zhang, Deqian Kong, Hongchuan Jiang, Meng Kou

**Affiliations:** 1 Northwest Institute of Nuclear Technology, Xi’an 710024, China; zhaoxiang_niu@outlook.com (Z.N.);; 2 School of Integrated Circuit Science and Engineering, University of Electronic Science and Technology of China, Chengdu 611731, China

**Keywords:** confined cavity explosion, temperature measurement, thermocouple sensor, system identification

## Abstract

This paper proposes a temperature measurement and system identification method for confined cavity explosions based on an improved type C thermocouple sensor. On the one hand, to address the extreme conditions caused by high-speed fragments and intense shock waves in an enclosed explosive environment, a thermocouple probe structure employing alloy strips of different widths with an alumina insulating layer in between is designed. By optimizing the strip width, the contact issues arising from edge-cutting burrs are effectively suppressed, thereby significantly enhancing the electrical insulation performance and overall reliability of the sensor. Additionally, a wedge-shaped alumina ceramic piece is designed to secure the thermocouple probe, further improving its structural stability under impact conditions. On the other hand, to tackle the highly nonlinear and multi-field coupled characteristics of the post-explosion temperature field, a system identification method based on the least square method is proposed. This method constructs a polynomial function in terms of radial distance and time variables, enabling effective reconstruction of the temperature field from limited measurement points. It provides a useful reference for understanding of the temperature distribution in confined cavity explosions and supports improved estimation of the temperature field. Finally, experimental results demonstrate that the improved sensor exhibits good survivability and measurement reliability under extreme explosive conditions. Meanwhile, the reconstructed temperature field model shows high fitting accuracy and good capability for describing the temperature distribution, confirming the effectiveness of the proposed identification method.

## 1. Introduction

Blasting technology, as a modern engineering technique, has been widely applied in various projects, such as underground tunnels, building demolition, and mining [1,2,3,4,5,6]. To effectively assess the impact of the explosion process on the surrounding environment and avoid potential hazards, the performance parameters characterizing the explosion process need to be accurately obtained, especially in confined spaces [7,8,9,10,11]. Compared to explosions in open air, the explosion process within a confined cavity involves unique physical phenomena such as shock wave reflection, complex wave system interactions, and confinement effects of high-temperature and high-pressure gases. These phenomena not only make the variation patterns of parameters like temperature and pressure during the explosion more complex but also impose higher demands on measurement techniques and analytical methods. Furthermore, extreme environmental conditions generated instantaneously during a confined cavity explosion, including intense shock waves, high-speed fragments, and high-temperature fireballs, pose severe challenges to the survivability of measurement sensors [12].

Temperature measurement is of significant importance for assessing damage effects in confined cavity explosions. Common temperature sensors can be broadly categorized into contact and non-contact types [13]. Compared to non-contact sensors like infrared sensors [14,15,16], contact sensors measure the object or medium itself directly through thermal conduction, offering higher measurement accuracy. Among existing contact sensors, thermocouple sensors have garnered widespread attention due to their advantages such as wide temperature measurement range and fast response time [17,18,19,20,21,22].

Specifically, for wire-type thermocouples, the thermal time constant is proportional to the square of the wire diameter due to its relationship with thermal mass. Therefore, reducing the diameter is a direct and effective method to achieve millisecond-level responses [23]. Advancing further, thin-film thermocouples with micro- or nano-scale junction thickness possess negligible thermal mass, enabling response times in the microsecond range [24,25]. However, both types of thermocouples are prone to damage under extreme conditions involving high-speed gas flow, high temperature, and high pressure due to their inherently low structural strength [24]. The fast-response surface thermocouples from NANMAC Corporation (Marlborough, MA, USA) are manufactured using special processes, with a maximum measurement temperature reaching 2300 °C [26,27]. NASA has used this company’s fast-response thermocouples for measuring the temperature of space capsules upon re-entry. Nevertheless, the conditions within a confined cavity explosion impose even more severe demands, combining intense shock waves, high-speed fragments, and rapid pressure transients that surpass the typical regimes of high-temperature aerodynamics. Crucially, there has been little discussion in the literature on systematically enhancing sensor survival rate and long-term reliability under this specific confluence of destructive forces. Therefore, to enable accurate temperature measurement in confined cavity explosions, it is imperative to research and develop a new class of fast-response thermocouple sensors that simultaneously achieve higher structural strength and greater impact resistance.

Furthermore, considering that the temperature changes in a confined cavity explosion exhibit strong nonlinearity and multi-field coupling characteristics—including tight coupling among chemical reactions, fluid motion, and heat conduction, as well as repeated reflection and superposition of shock waves within the confined space—the system dynamics present a high degree of complexity. This makes it difficult to obtain a post-explosion temperature model through physical analysis or by establishing relevant state equations. Therefore, it is necessary to conduct identification of the temperature field inside a confined cavity explosion to enhance the analysis capability of this extreme physical process of post-explosion temperature changes.

Addressing the aforementioned issues, this paper proposes a method for temperature measurement and system identification in confined cavity explosions based on an improved Type C thermocouple sensor. An improved Type C thermocouple temperature sensor with impact-resistant and fast-response performance, designed for use in confined cavity explosions, is presented. A systematic analysis, modeling, and identification of the post-explosion temperature field model are conducted. The main contributions of this paper are as follows:

(1) An improved Type C thermocouple sensor is proposed. On the one hand, considering the cutting burrs at the edges of the alloy thin strips, a thermocouple probe structure is designed using alloy thin strips of different widths with an alumina insulating layer in between, effectively improving electrical insulation performance. On the other hand, a wedge-shaped alumina ceramic plate is designed to secure the thermocouple probe, further enhancing the sensor’s impact resistance in explosive environments, thereby aiding sensor survival and reliable temperature measurement under extreme conditions.
(2) This paper establishes a polynomial function model related to fractional powers of radial distance and logarithmic time to describe the complex temperature change process under confined cavity explosion conditions. The least square method is employed to solve for the analytical expression of the post-explosion temperature field model. This identification method can effectively capture the temperature change trend, facilitating the reconstruction of the temperature field from limited measurement points and providing enhanced analysis and visualization of the post-explosion thermal behavior.

## 2. The Proposed Measurement and Identification Scheme

**Figure 1:**
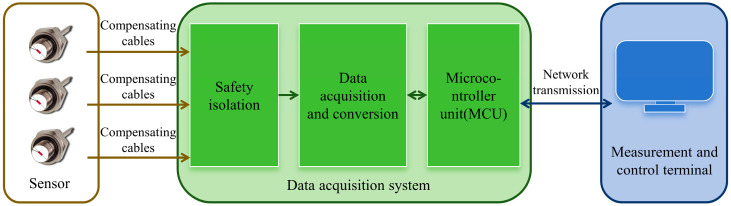
Structure of the temperature measurement system for the confined cavity explosion field.

The overall structure of the temperature measurement system for the confined cavity explosion field is shown in Figure 1. First, the thermocouple sensor directly generates a thermoelectric voltage signal through the Seebeck effect in response to the temperature difference. This voltage is transmitted to the backend data acquisition unit via compensating cables. Next, after processing steps such as safety isolation, analog-to-digital conversion, and data filtering, the obtained digital signal is transmitted to the measurement and control terminal through a communication network. Finally, the data is recorded, processed, and displayed by a computer, thereby obtaining the temperature value inside the cavity after the explosion.

Specifically, the data acquisition system employed in this study consists of a UEILogger600 data acquisition chassis (United Electronic Industries, Inc., Norwood, MA, USA) equipped with a DNx AI 212 analog input module (United Electronic Industries, Inc., Norwood, MA, USA). The system is configured with a sampling rate of 1.5 kHz and a 24-bit analog-to-digital conversion resolution. Anti-aliasing filtering is applied at a cutoff frequency corresponding to 47.6% of the sampling rate to prevent signal aliasing and ensure data fidelity. For temperature measurements using Type C thermocouples, the system provides a maximum measurement error of ±0.6 °C, guaranteeing high accuracy for the experimental data obtained in this work.

To enhance the understanding of the phenomenology of confined space explosions, it is necessary to obtain an appropriate temperature model and conduct theoretical analysis. However, the post-explosion temperature field inside a confined cavity is governed by strongly coupled, multi-scale physical processes, including shock wave propagation/reflection and turbulent afterburning of detonation products, as well as heat transfer between the gas and the cavity walls [28,29]. First-principles modeling of these phenomena requires solving a system of partial differential equations with detailed boundary conditions that are rarely available in engineering practice [29]. Consequently, an analytical solution to the spatiotemporal temperature distribution is practically unattainable under realistic explosive loading.

To circumvent this difficulty, a data-driven reduced-order modeling strategy is adopted to reconstruct the post-explosion temperature field model in this paper. In a homogeneous medium, the temperature field formed by an explosion exhibits a hemispherical shape spreading outward from a point, presenting a significant radially symmetric distribution. Under ideal conditions, the temperature distribution at positions with the same radius is highly consistent. Then, the temperature field can be treated as an unknown nonlinear mapping T=f(r,t), where r is the radial distance from the explosion center and t is the time after detonation. Although the temperature field is highly transient in the early stage, within the finite spatiotemporal window covered by the present measurements, the temperature data exhibit smooth variations in both space and time. This empirical observation permits a polynomial approximation—essentially a local Taylor expansion in the variables r and t. Accordingly, the explosion temperature field model can be constructed as follows:

(1) $$\begin{array}{ll} T\left(r,t\right) & =\;\sum_{i=0}^{m}\sum_{j=0}^{i}a_{ij}r^{j}t^{i-j}\\ & =\;a_{0}+a_{10}t+a_{11}r+a_{20}t^{2}+a_{21}rt+a_{22}r^{2}+\cdots \\ & +a_{m0}t^{m}+\cdots +a_{m(m-1)}r^{m-1}t+a_{mm}r^{m} \end{array}$$

where m denotes the highest order of the polynomial function; $a_{0},a_{10},a_{11},a_{20},\ldots ,a_{mm}$ represent the polynomial coefficients to be determined. Considering that the post-explosion temperature curve approximately exhibits exponential rise and decay over time [30], in order to improve model fitting accuracy while reducing the polynomial order, the temperature function T(r,t) related to radial distance and time is rewritten in this paper as T(r,lnt). Furthermore, to effectively suppress potential temperature overfitting caused by the large numerical range of the radial distance *r* and its higher-order terms, and to better capture the nonlinear decay characteristics of the temperature field with distance, the model T(r,lnt) is further reformulated in this study as $T(\sqrt[{n}]{r},lnt)$. Herein, the parameter *n* (*n* > 0) governs the sharpness of the decay rate, and its specific value will be optimally determined from the experimental data through the subsequent system identification procedure. Consequently, a polynomial function model describing temperature variation with $\sqrt[{n}]{r}$ and lnt is constructed as follows:

(2) $$\begin{array}{ll} T\left(\sqrt[{n}]{r},lnt\right) & =\;\sum_{i=0}^{m}\sum_{j=0}^{i}a_{ij}{\left(\sqrt[{n}]{r}\right)}^{j}{\left(lnt\right)}^{i-j}\\ & =\;a_{0}+a_{10}lnt+a_{11}\sqrt[{n}]{r}+a_{20}{\left(lnt\right)}^{2}+a_{21}\sqrt[{n}]{r}lnt+a_{22}{\left(\sqrt[{n}]{r}\right)}^{2}+\cdots \\ & +a_{m0}{\left(lnt\right)}^{m}+\cdots +a_{m(m-1)}{\left(\sqrt[{n}]{r}\right)}^{m-1}lnt+a_{mm}{\left(\sqrt[{n}]{r}\right)}^{m} \end{array}$$

In the constructed polynomial function (2), the constant term $a_{0}$ can be regarded as the temperature baseline under specific reference conditions; the terms $a_{11}\sqrt[{n}]{r},a_{22}{\left(\sqrt[{n}]{r}\right)}^{2},\ldots ,\;a_{mm}{\left(\sqrt[{n}]{r}\right)}^{m}$ describe the linear and higher-order variation trends of temperature with $\sqrt[{n}]{r}$; the terms $a_{10}lnt,a_{20}{\left(lnt\right)}^{2},\ldots ,a_{m0}{\left(lnt\right)}^{m}$ represent the linear and higher-order variation trends of temperature with lnt, respectively; furthermore, considering that the rate of temperature change over time may differ at positions with varying radial distances from the explosion point, terms such as $a_{21}\sqrt[{n}]{r}lnt,a_{31}\sqrt[{n}]{r}{\left(lnt\right)}^{2},a_{32}{\left(\sqrt[{n}]{r}\right)}^{2}lnt,\ldots ,a_{m(m-1)}{\left(\sqrt[{n}]{r}\right)}^{m-1}lnt$ can characterize the coupled influence of radial distance and time on temperature.

When conducting temperature measurement in a confined space explosion field, it is necessary to deploy temperature sensors at different positions inside the explosion field. This paper plans to set up three temperature measurement points at different radial distances within the explosion field. The distances from these three measurement points to the explosion center are denoted as $r_{1},r_{2},r_{3}$, respectively. Thus, the fitting function for temperature change over time at each measurement point can be written as:

(3) $$\begin{array}{ll} T_{1}\left(lnt\right) & =\;\sum_{i=0}^{m}\sum_{j=0}^{i}a_{ij}{{(\sqrt[{n}]{r}}_{1})}^{j}{\left(lnt\right)}^{i-j}\\ & =\;a_{0}+a_{10}lnt+a_{11}{\sqrt[{n}]{r}}_{1}+a_{20}{\left(lnt\right)}^{2}+..+a_{mm}{{(\sqrt[{n}]{r}}_{1})}^{m} \end{array}$$

(4) $$\begin{array}{ll} T_{2}\left(lnt\right) & =\;\sum_{i=0}^{m}\sum_{j=0}^{i}a_{ij}{{(\sqrt[{n}]{r}}_{2})}^{j}{\left(lnt\right)}^{i-j}\\ & =\;a_{0}+a_{10}lnt+a_{11}{\sqrt[{n}]{r}}_{2}+a_{20}{\left(lnt\right)}^{2}+..+a_{mm}{{(\sqrt[{n}]{r}}_{2})}^{m} \end{array}$$

(5) $$\begin{array}{ll} T_{3}\left(lnt\right) & =\;\sum_{i=0}^{m}\sum_{j=0}^{i}a_{ij}{{(\sqrt[{n}]{r}}_{3})}^{j}{\left(lnt\right)}^{i-j}\\ & =\;a_{0}+a_{10}lnt+a_{11}{\sqrt[{n}]{r}}_{3}+a_{20}{\left(lnt\right)}^{2}+..+a_{mm}{{(\sqrt[{n}]{r}}_{3})}^{m} \end{array}$$

Assuming that temperatures are measured at a total of *N* time points, $t_{1},t_{2},\ldots t_{N}$, during the experiment, then a total of 3*N* data results are obtained from the three measurement points. Denoting the vector of measured temperature data as Y, the fitted temperature data from the constructed explosion field model is denoted as $\hat{Y}$, which is written as a 3*N* × 1 column vector as follows:

(6) $$\begin{array}{l} \hat{Y}=[T_{1}({lnt}_{1}),T_{1}({lnt}_{2}),\ldots ,T_{1}({lnt}_{N}),T_{2}(lnt_{1}),\\ T_{2}({lnt}_{2}),\ldots ,T_{2}({lnt}_{N}),T_{3}({lnt}_{1}),T_{3}({lnt}_{2}),\ldots ,T_{3}(lnt_{N}){]}^{T} \end{array}$$

By combining Equations (2)–(6), $\hat{Y}$ can be expressed in the following matrix form:

(7) $$\hat{Y}=X\beta$$

where

(8) $$\beta ={\left[a_{0},a_{10},a_{11},a_{20},\ldots ,a_{mm}\right]}^{T}$$

(9) $$X=\left[\begin{array}{c} 1,{lnt}_{1},{\sqrt[{n}]{r}}_{1},{\left({lnt}_{1}\right)}^{2},\ldots ,{\left({\sqrt[{n}]{r}}_{1}\right)}^{m}\\ 1,{lnt}_{2},{\sqrt[{n}]{r}}_{1},{\left({lnt}_{2}\right)}^{2},\ldots ,{\left({\sqrt[{n}]{r}}_{1}\right)}^{m}\\ \vdots \\ 1,{lnt}_{N},{\sqrt[{n}]{r}}_{1},{\left({lnt}_{N}\right)}^{2},\ldots ,{\left({\sqrt[{n}]{r}}_{1}\right)}^{m}\\ 1,{lnt}_{1},{\sqrt[{n}]{r}}_{2},{\left({lnt}_{1}\right)}^{2},\ldots ,{\left({\sqrt[{n}]{r}}_{2}\right)}^{m}\\ 1,{lnt}_{2},{\sqrt[{n}]{r}}_{2},{\left({lnt}_{2}\right)}^{2},\ldots ,{\left({\sqrt[{n}]{r}}_{2}\right)}^{m}\\ \vdots \\ 1,{lnt}_{N},{\sqrt[{n}]{r}}_{2},{\left({lnt}_{N}\right)}^{2},\ldots ,{\left({\sqrt[{n}]{r}}_{2}\right)}^{m}\\ 1,{lnt}_{1},{\sqrt[{n}]{r}}_{3},{\left({lnt}_{1}\right)}^{2},\ldots ,{\left({\sqrt[{n}]{r}}_{3}\right)}^{m}\\ 1,{lnt}_{2},{\sqrt[{n}]{r}}_{3},{\left({lnt}_{2}\right)}^{2},\ldots ,{\left({\sqrt[{n}]{r}}_{3}\right)}^{m}\\ \vdots \\ 1,{lnt}_{N},{\sqrt[{n}]{r}}_{3},{\left({lnt}_{N}\right)}^{2},\ldots ,{\left({\sqrt[{n}]{r}}_{3}\right)}^{m} \end{array}\right]$$

According to the principle of the least square method, the optimal value for the undetermined parameter vector β is the one that minimizes the objective loss function

(10) $$J\left(\beta \right)={\left\|Y-\hat{Y}\right\|}^{2}={\left\|Y-X\beta \right\|}^{2}={\left(Y-X\beta \right)}^{T}\left(Y-X\beta \right)$$

By simplifying the objective function $J\left(\beta \right)$, we obtain

(11) $$J\left(\beta \right)=\beta^{T}X^{T}X\beta -\beta^{T}X^{T}Y-Y^{T}X\beta +Y^{T}Y$$

To find the extremum of the objective function, we take the derivative of Equation (11) and set it equal to zero:

(12) $$\frac{\partial }{\partial \beta }J\left(\beta \right)=2X^{T}X\beta -2X^{T}Y=0$$

Solving Equation (12) gives

(13) $$\beta ={\left(X^{T}X\right)}^{-1}X^{T}Y$$

Thus, the values of the fitting model parameters $a_{0},a_{10},a_{11},a_{20},\ldots ,a_{mm}$ in Equation (2) are obtained. In summary, the constructed polynomial model (2) fits the explosion temperature field, which exhibits strong nonlinearity and multi-field coupling characteristics, into a bivariate function related to temperature and time. It effectively characterizes the dynamic evolution and spatial distribution characteristics of post-explosion temperature, providing assistance for assessing explosion damage effects and ensuring experimental safety.

## 3. The Proposed Improved Type C Thermocouple Sensor

### 3.1. Temperature Measurement Principle of Thermocouple Sensors

**Figure 2:**
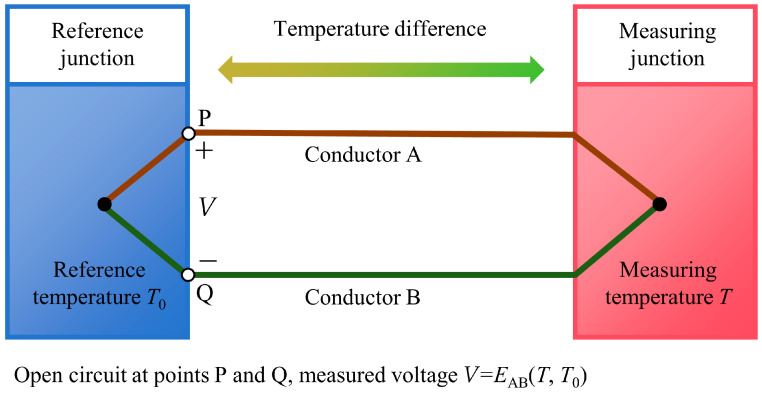
Schematic diagram of a typical thermocouple measurement circuit based on Seebeck effect.

A thermocouple is a sensor that converts temperature differences into electromotive force (EMF) signals based on the Seebeck effect [31,32,33]. It is widely used in industrial temperature measurement due to its simple structure, convenient manufacturing, wide measurement range, fast response, and vibration resistance. The theoretical foundation and historical development of this thermoelectric principle have been thoroughly documented in classical works [34,35,36,37]. For practical application, the temperature-EMF relationship of standardized thermocouples, including Type C, is defined by international standards such as ASTM E230 [38]. Figure 2 shows a schematic diagram of a typical thermocouple measurement circuit based on this effect. Specifically, when two conductors (or semiconductors) A and B of different materials are joined end-to-end to form a closed loop, a small electromotive force is generated in the loop if a temperature difference exists between the two junctions: one end is the measuring junction (hot junction, temperature *T*) and the other is the reference junction (cold junction, temperature *T*_0_). For a given pair of materials A and B, the total thermoelectric electromotive force generated is $E_{AB}(T,T_{0})=f(T)-f(T_{0})$. This implies that when the reference junction temperature *T*_0_ is kept constant, the thermoelectromotive force $E_{AB}$ becomes a single-valued function of the measuring junction temperature *T*.

In actual measurements, the closed loop is opened at points P and Q, and the open-circuit voltage *V* between these points is recorded by the data acquisition system. Under ideal open-circuit conditions, the measured voltage *V* is numerically equivalent to the thermoelectric electromotive force $E_{AB}(T,T_{0})$. Then the measuring junction temperature *T* can be determined from the measured voltage *V*. The data acquisition system employed in this study is detailed in Section 2.

### 3.2. Structural Design of the Improved Type C Thermocouple Sensor

**Figure 3:**
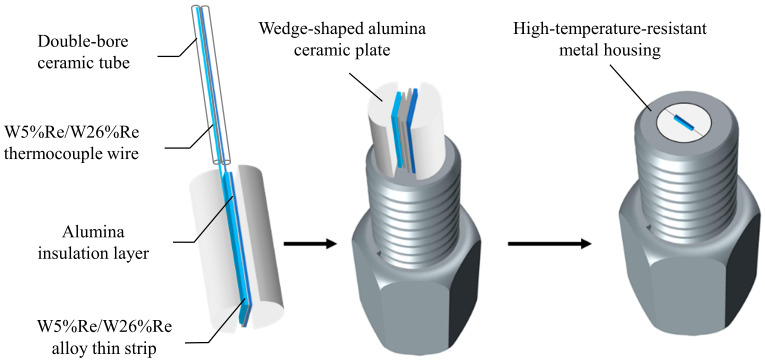
Schematic diagram of the designed thermocouple structure.

The designed thermocouple sensor mainly consists of W5%Re/W26%Re alloy thin strip, alumina insulation layer, W5%Re/W26%Re thermocouple wire, wedge-shaped alumina ceramic plate, double-bore ceramic tube, and high-temperature-resistant metal housing, as shown in Figure 3. W5%Re and W26%Re are used as the two electrode materials for the Type C thermocouple, with a temperature measurement range of 0⁓2315 °C. The high-temperature-resistant metal housing is made of a molybdenum alloy. Its extremely high melting point and superior impact resistance ensure reliable protection for the internal sensitive components against the transient extreme temperature and pressure generated by a confined cavity explosion.

**Figure 4:**
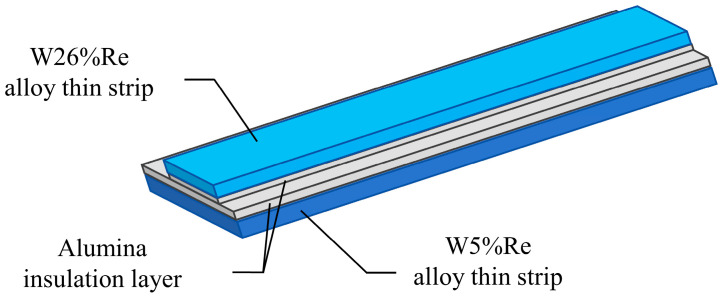
Schematic diagram of contact between W5%Re/W26%Re alloy thin strips.

The temperature-sensitive end of the thermocouple adopts a “sandwich” structure consisting of a W5%Re alloy thin strip and a W26%Re alloy thin strip with an alumina insulation layer in between, as shown in Figure 4. It is noteworthy that during the preparation of the alloy thin strips, the cutting process makes it difficult to ensure perfectly smooth edges, resulting in certain burrs at the strip edges. If two alloy thin strips of the same width are overlapped, the cutting burrs at the edges could easily come into contact and conduct electricity, leading to poor insulation performance at the edges. To address this issue, this paper uses two alloy thin strips of different widths, W5%Re and W26%Re, and overlaps them, as shown in Figure 4. This avoids direct contact between the edge burrs of the two alloy strips, thereby achieving a better electrical insulation effect and enhancing the measurement stability of the sensor.

**Figure 5:**
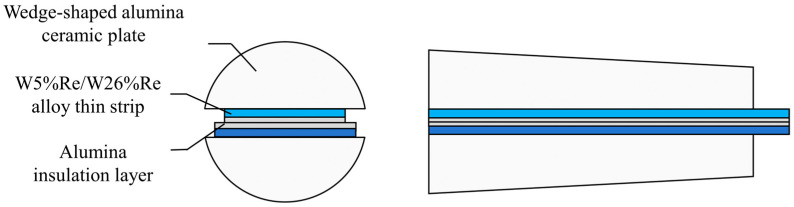
Schematic of the wedge-shaped alumina ceramic plate structure.

To withstand the intense airflow shock and high-speed fragment impact under confined cavity explosion conditions and improve the sensor’s survival rate and reliability, the wedge-shaped alumina ceramic plate, as shown in Figure 5, is designed to secure the thermocouple probe. The wedge-shaped alumina ceramic plate features a structure that is wider at the front end and narrower at the rear end, with its front-end diameter slightly larger than that of the outermost high-temperature-resistant metal housing of the sensor. When the thermocouple probe is clamped between two wedge-shaped ceramic plates and inserted together into the metal housing, the ductility of the metal is utilized to achieve a tight fixation of the thermocouple probe. This effectively enhances the sensor’s sealing performance and impact resistance, thereby ensuring the consistency and accuracy of the measurement data.

The hot junction of the designed Type C thermocouple sensor is the connection point between the W5%Re/W26%Re thin strips exposed on the hot end surface, achieved by grinding to create a weak connection for electrical conduction between the two alloy strips. The hot junction produced by this process has a very small volume and consequently a very low heat capacity, which can significantly reduce the sensor’s response time. After high-temperature testing, the surface of the hot junction is prone to oxidation. At this point, it can be re-ground to remove the surface oxide layer, enabling hot junction renewal and sensor reuse. The connection part between the thermocouple probe and the rear-end plug uses W5%Re/W26%Re thermocouple wires of the same material as the W5%Re/W26%Re alloy thin strips, providing a certain temperature compensation effect. The two thermocouple wires are placed inside a double-bore ceramic tube to ensure electrical insulation.

## 4. Calibration and Temperature Measurement Uncertainty

### 4.1. System Calibration

A dynamic temperature measurement system is established using a Type C thermocouple and a UEILogger600 data acquisition instrument. The thermal response time is calculated based on 63.2% rise time of temperature step input induced by laser excitation. The detailed calibration results from three repeated measurements are summarized in Table 1.

Considering that the thickness of the thermocouple alloy strip requires a reasonable trade-off between mechanical strength and response time: a larger thickness brings stronger mechanical robustness but longer thermal response time, while a smaller thickness enables faster response but weaker structural strength. In the high-impact explosion environment of this study, an extremely thin alloy strip cannot be used to ensure structural integrity and reliable temperature measurement. Therefore, an alloy strip with a thickness of 0.1 mm is adopted, limiting the thermal response time to approximately 13 ms. Constrained by this engineering trade-off, extremely transient and microsecond-level temperature jumps are difficult to capture completely without distortion. This is a typical limitation of thermocouple sensors in strong shock and rapidly varying thermal environments. This study mainly focuses on improving the measurement reliability of the thermocouple and designing the spatiotemporal distribution reconstruction method of temperature field. Within this research framework, the dynamic response characteristics of the system can basically support the core content of this work.

### 4.2. Uncertainty Analysis

According to the GUM (Guide to the Expression of Uncertainty in Measurement) [39], the measurement uncertainty is evaluated assuming a uniform distribution with a coverage factor $k=\sqrt{3}$. The main uncertainty sources are the Type C thermocouple and the data acquisition module.

For the Type C thermocouple, within its valid operating range of 425–2315 °C, the maximum allowable error is ±1.0% of the measured temperature. Its relative standard uncertainty is

(14) $$u_{rel,1}=\frac{1.0\%}{\sqrt{3}}=0.577\%$$

For the data acquisition module with a maximum error of ±0.6 °C mentioned in Section 2, its relative standard uncertainty at typical temperature of 1000 °C is

(15) $$u_{rel,2}=\frac{0.6}{1000\sqrt{3}}=0.035\%$$

The relative combined standard uncertainty is calculated by the root-sum-square method as follows:

(16) $$u_{rel}=\sqrt{{u_{rel,1}}^{2}+{u_{rel,2}}^{2}}=\sqrt{0.577^{2}+0.035^{2}}=0.578\%$$

It can be concluded that the Type C thermocouple provides the primary contribution to the overall temperature measurement uncertainty, while the influence of the data acquisition module is relatively small.

## 5. Results and Discussion

**Figure 6:**
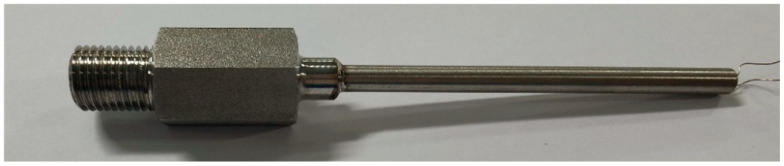
Physical photograph of the designed improved Type C thermocouple sensor.

**Figure 7:**
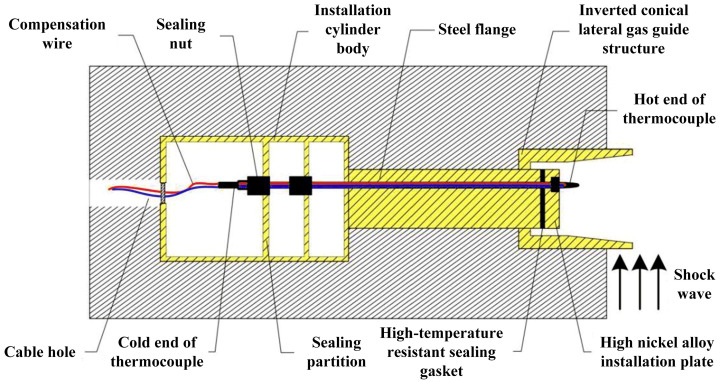
Basic structure of the temperature sensor protective device.

In this paper, the explosion experiments in a confined cavity are designed and carried out. The confined cavity has dimensions of 3 m×2 m×10 m, and TNT is used as the explosive with a charge mass of 200 kg. The temperature measurement system for the explosion field, as shown in Figure 1, is employed, with the designed improved Type C thermocouple sensor (see Figure 6) serving as the core temperature measurement device for this experiment. To enhance the survivability of the sensor, a sensor installation and protection structure is designed, as shown in Figure 7. The front end of this protective structure is exposed to the cavity to improve the sensitivity of the temperature sensor, while the cable for communication with the data acquisition system is led out from the rear end of the device.

**Figure 8:**
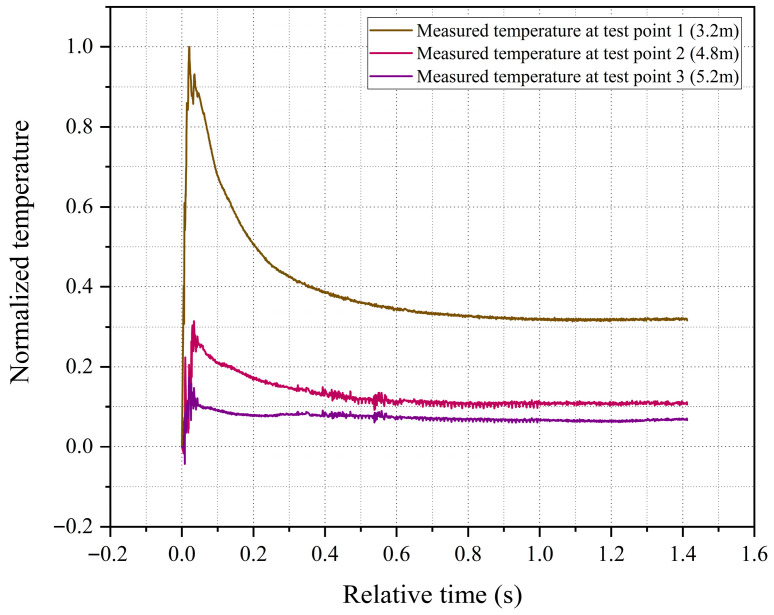
Post-explosion temperature measurement curves for the three test points.

This paper placed thermocouple temperature sensors and their protective devices at three test points (labeled 1, 2, and 3) with different radial positions to conduct research on explosion temperatures. The radial distances from test points 1, 2, and 3 to the explosion center are 3.2 m, 4.8 m, and 5.2 m, respectively. Figure 8 shows the post-explosion temperature measurement curves for the three test points, where the measured temperature values have been normalized. Additionally, the uncertainty of temperature measurement is analyzed in detail in Section 4.2. From the experimental measurement results, the temperature curves obtained at all measurement points are continuous and stable, without significant signal interruption or abnormal fluctuation. This fully demonstrates that the sensor maintains robust structural survivability and operational reliability even under extreme explosive conditions. The measured temperatures at all three test points rose to their peak values around 20 to 30 ms after the explosion, and the peak temperatures showed a generally negative correlation with the distance from the explosion center.

Next, based on the polynomial function (2) constructed in Section 2, which is related to fractional powers of radial distance and logarithmic time, and combined with the derived calculation formula (13) for the fitting parameters $a_{0},a_{10},a_{11},a_{20},\ldots ,a_{mm}$, the post-explosion cavity temperature field model is reconstructed. To determine the appropriate polynomial parameters for the temperature field model, this paper compares the accuracy of fitting models with different polynomial orders *m* and distance attenuation parameters *n*, respectively. The goodness of fit is selected as the evaluation metric for fitting accuracy, and its expression is:

(17) $$R^{2}=1-\frac{\sum_{i=1}^{4N}{\left(T_{i}-{\hat{T}}_{i}\right)}^{2}}{\sum_{i=1}^{4N}{\left(T_{i}-\bar{T}\right)}^{2}}$$

where $T_{i},{\hat{T}}_{i},\bar{T}$ represent the sensor-measured temperature data, the fitted temperature data from the model, and the mean of the sensor-measured temperatures, respectively. The value of $R^{2}$ ranges from 0 to 1, and a larger value signifies higher model fitting accuracy. The goodness-of-fit values for temperature field models with different polynomial orders *m* and distance attenuation parameters *n* (both ranging from 1 to 5) are listed in Table 2.

From Table 2, it can be observed that the goodness of fit of the model varies very little with the distance attenuation parameter *n*, which is mainly attributed to the relatively small absolute values of the radial distances of the explosion measurement points adopted in this study. However, to ensure the universality of the designed fitting model and address the temperature field reconstruction problems that may arise under lager explosive masses and longer measurement distances in the future, this distance attenuation term still needs to be retained. After comprehensively balancing the fitting accuracy, computational efficiency, and long-term universality, this work selects a polynomial model with *m* = 4 and *n* = 4 to characterize the spatiotemporal evolution of post-explosion temperature. The choice of *n* = 4 not only effectively compresses the radial distance scale to stabilize numerical performance for future large-scale scenarios but also maintains an excellent goodness-of-fit that is nearly identical to smaller *n* values, ensuring no sacrifice in data fitting accuracy.

**Figure 9:**
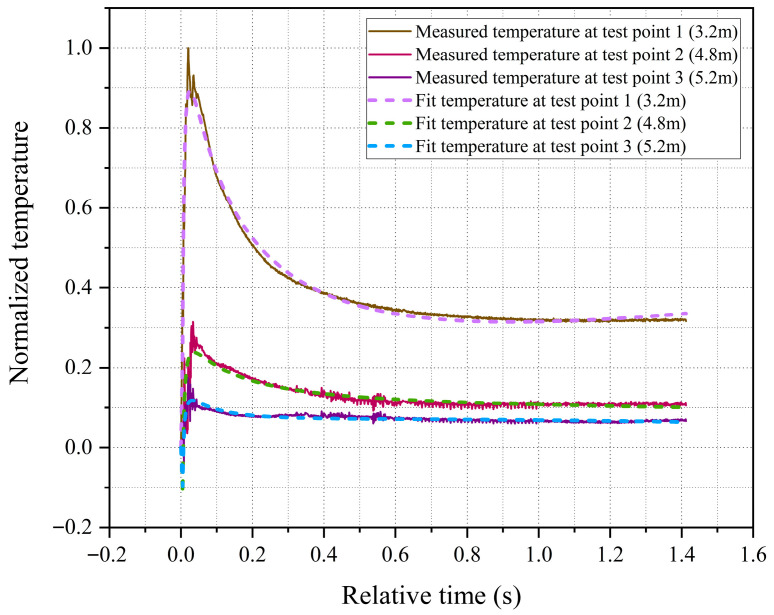
Comparison of measured and fitted temperature data.

Subsequently, according to the least square parameter calculation formula (13), the fitting parameters $a_{0},a_{10},a_{11},a_{20},\ldots ,a_{44}$ is calculated, and the values are shown in Table 3. The model temperature values output by the fitted model are compared with the experimentally measured temperature values, and the corresponding comparison results are plotted in Figure 9.

**Figure 10:**
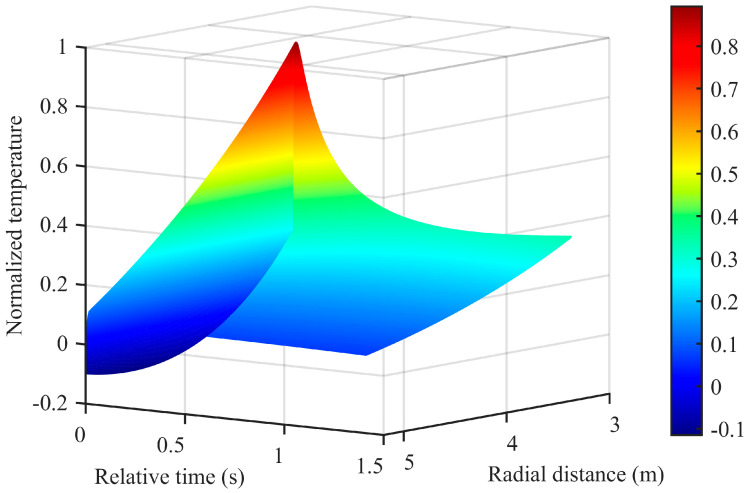
Temperature field model related to radial distance and time.

As can be seen from the curves in Figure 9, the fitted model closely coincides with the measured temperature data, effectively reflecting the post-explosion temperature change process, and the goodness of fit reaches 0.9943. The complete fitted temperature field model related to radial distance and time is displayed in Figure 10.

**Figure 11:**
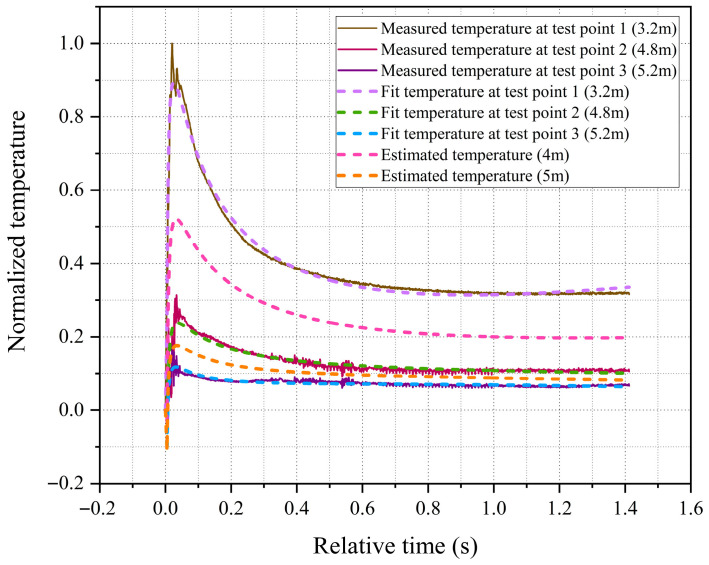
Estimated temperature curves at radial distances of 4 m and 5 m, respectively.

Furthermore, constrained by the cost of deploying sensors and protective devices, this paper collected temperature data from only three test points. However, in practical engineering applications, it is often necessary to obtain temperature data at different locations (especially in areas where sensor deployment is difficult). In such cases, the fitted temperature field model provides a means for reasonable extrapolation. To assess this extrapolation capability, two sensor-free locations were selected for analysis, as shown in Figure 11. The first is at a radial distance of 4 m, situated between test point 1 and test point 2. The estimated post-explosion temperature curve at this location peaks around 30 ms, with its peak value falling between the measured peak temperatures of the two adjacent test points. This result is consistent with the observed negative correlation between peak temperature and distance from the explosion center. The second location is at a radial distance of 5 m, between test point 2 and test point 3. The estimated peak temperature at this position is lower than the measured value of test point 2 and higher than that of test point 3, consistent with the expected spatial variation of peak temperature. Collectively, these two extrapolation examples validate the model’s capability to provide reasonable temperature estimates at unmeasured locations.

**Figure 12:**
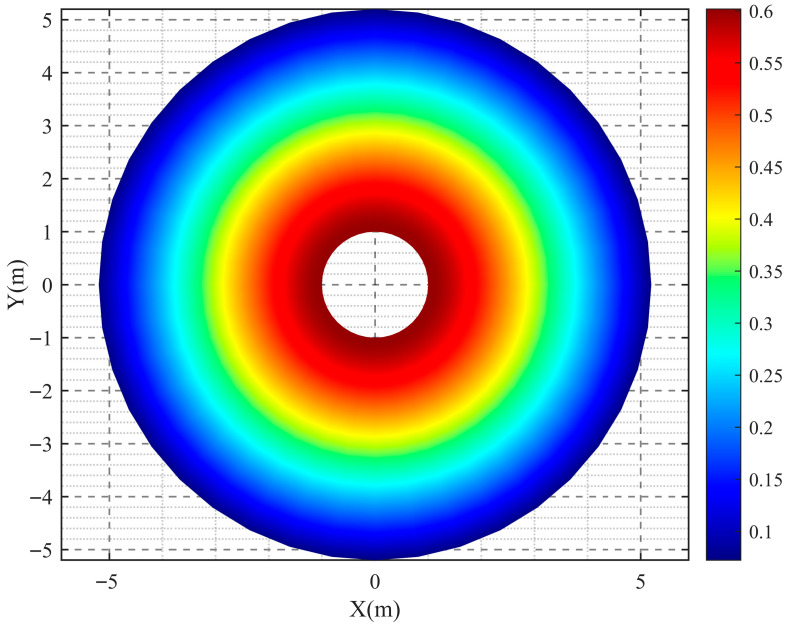
Spatial distribution of the temperature field at *t* = 0.5 s.

**Figure 13:**
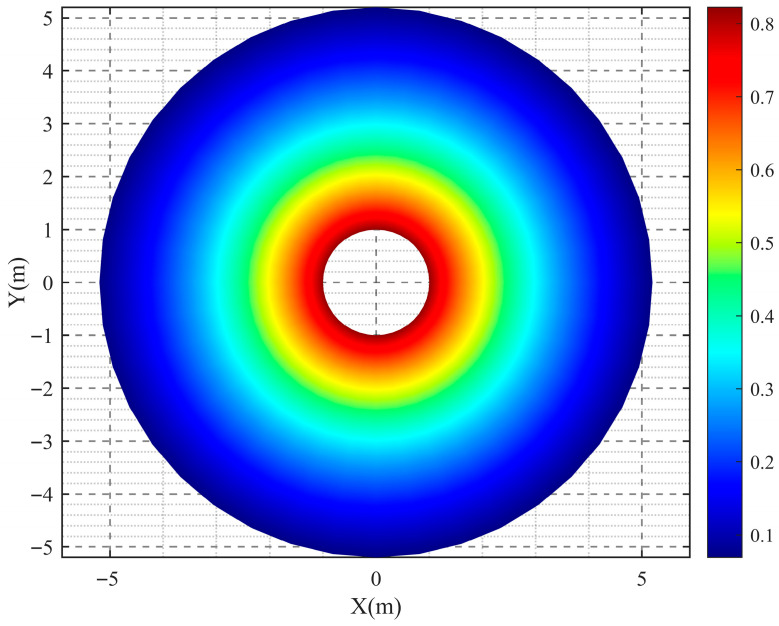
Spatial distribution of the temperature field at *t* = 1 s.

Moreover, Figure 12 and Figure 13 present the simulated spatial distributions of the temperature field at time instants *t* = 0.5 s and *t* = 1 s, respectively. Due to the strong interference from shock waves and high-speed fragments near the explosion center, it is difficult to directly acquire data using sensors in this region; meanwhile, extrapolation of data in this area also suffers from accuracy limitations. Therefore, this study mainly establishes a temperature field distribution model for regions beyond 1 m from the explosion center. Future work will focus on continuously improving the survivability of sensors under confined cavity explosion conditions and conducting in-depth investigation into the temperature variation patterns in the vicinity of the explosion center.

## 6. Conclusions

In conclusion, to meet the temperature measurement requirements in confined cavity explosion environments, this study designs an improved Type C thermocouple sensor. Its probe utilizes a structure of alloy thin strips of different widths with an alumina insulating layer in between and is secured using wedge-shaped alumina ceramic plates. This enhances the sensor’s insulation performance and impact resistance, facilitating sensor survival and effective temperature measurement in confined cavity explosion settings. Moreover, based on the experimentally acquired temperature data, a polynomial temperature field model related to radial distance and time is established and identified via the least-squares method. The close agreement between the fitted model and the measured data demonstrates the effectiveness of the proposed identification approach. This temperature field model and the associated identification method enable effective reconstruction of the post-explosion cavity temperature field using only limited measurement points, offering a practical tool for analyzing and visualizing of temperature evolution after an explosion.

## Figures and Tables

**Table 1:** Thermal response time calibration results.

No. of Measurement	Pre-Step Voltage/mV	Post-Step Voltage/mV	Thermal Response Time *τ*_0.632_/ms	Average Response Time *τ*_ave_/ms
1	0.03	1.03	13.00	13.00
2	0.13	1.05	14.00	
3	0.11	1.05	12.00	

**Table 2:** Goodness-of-fit values of temperature field models for different polynomial orders *m* and distance attenuation parameters *n*.

Values of Goodness of Fit	*m* = 1	*m* = 2	*m* = 3	*m* = 4	*m* = 5
*n* = 1	0.8523	0.9528	0.9776	0.9944	0.9961
*n* = 2	0.8528	0.9529	0.9777	0.9943	0.9961
*n* = 3	0.8528	0.9529	0.9777	0.9943	0.9961
*n* = 4	0.8529	0.9529	0.9777	0.9943	0.9961
*n* = 5	0.8529	0.9529	0.9777	0.9943	0.9961

**Table 3:** Fitted parameter values for the explosion temperature field model.

Values	(ln*t*)^0^	(ln*t*)^1^	(ln*t*)^2^	(ln*t*)^3^	(ln*t*)^4^
${\left(\sqrt[{4}]{r}\right)}^{0}$	$a_{0}$ = 0.5877	$a_{10}$ = 6.1498	$a_{20}$ = 3.9213	$a_{30}$ = 0.1859	$a_{40}$ = −0.0016
${\left(\sqrt[{4}]{r}\right)}^{1}$	$a_{11}$ = 2.1397	$a_{21}$ = −6.8957	$a_{31}$ = −4.7049	$a_{41}$ = −0.1293	
${\left(\sqrt[{4}]{r}\right)}^{2}$	$a_{22}$ = −1.9071	$a_{32}$ = 0.6575	$a_{42}$ = 1.3909		
${\left(\sqrt[{4}]{r}\right)}^{3}$	$a_{33}$ = −0.3342	$a_{43}$ = 0.8001			
${\left(\sqrt[{4}]{r}\right)}^{4}$	$a_{44}$ = 0.3365				

## Data Availability

The data presented in this study are available on request from the corresponding author.
